# Spatio-Temporal Resource Mapping for Intensive Care Units at Regional Level for COVID-19 Emergency in Italy

**DOI:** 10.3390/ijerph17103344

**Published:** 2020-05-12

**Authors:** Pietro Hiram Guzzi, Giuseppe Tradigo, Pierangelo Veltri

**Affiliations:** Department of Surgical and Medical Sciences, University of Catanzaro, 88100 Catanzaro, Italy; gtradigo@unicz.it (G.T.); veltri@unicz.it (P.V.)

**Keywords:** COVID-19, prediction of infected, data analysis

## Abstract

COVID-19 is a worldwide emergency since it has rapidly spread from China to almost all the countries worldwide. Italy has been one of the most affected countries after China. North Italian regions, such as Lombardia and Veneto, had an abnormally large number of cases. COVID-19 patients management requires availability of sufficiently large number of Intensive Care Units (ICUs) beds. Resources shortening is a critical issue when the number of COVID-19 severe cases are higher than the available resources. This is also the case at a regional scale. We analysed Italian data at regional level with the aim to: (i) support health and government decision-makers in gathering rapid and efficient decisions on increasing health structures capacities (in terms of ICU slots) and (ii) define a geographic model to plan emergency and future COVID-19 patients management using reallocating them among health structures. Finally, we retain that the here proposed model can be also used in other countries.

## 1. Introduction

COVID-19 [1] is caused by the SARS-CoV-2 virus and belongs to the Coronaviridæ family, which contains many other viruses. Only seven of which are known to be responsible for human diseases, e.g., 229E, NL63, OC43, HKU1, MERS-CoV, SARS-CoV, and SARS-CoV-2 [2,3].

The virus diffused with a surprisingly fast pace, and in one month putting under stress the healthcare resources worldwide, starting from China. Italy was the first European country affected by the virus. The high spreading rate and the absence of tailored therapies and vaccines determine a relatively high mortality rate that has been controlled by blocking the virus spreading with severe mobility restrictions to the people of the infected regions [3].

By the end of March, while the situation in China seems to be under control, the virus is rapidly growing in other countries [4,5]. With different time scales, other countries such as the USA, France, Spain and North Europe reacted by implementing containment measures. The virus has an initial exponential diffusion which requires: (i) home quarantine for low symptoms, (ii) hospitalisation for part of them and, (iii) hospitalisation in ICUs requiring respiratory support for severe ones.

In some cases, COVID-19 causes severe pneumonia, especially in the presence of co-morbidities [6], thus patients need hospitalization in ICU where respiratory support (such as mechanical ventilators) are required to keep them alive [7].

We focus on a disease diffusion model which helps predicting ICU resources, for the Italian emergency. The model is general enough to foresee its adoption also in other countries. It also scales well at a regional or sub-regional level.

## 2. Materials and Methods

Data.

All data used in this work are provided by Italian Government on a publicly available web site https://github.com/pcm-dpc/COVID-19 under licence CC-BY-4.0.

## 3. Results and Discussion

We start from the analysis of epidemiological data from Wuhan city (China, Hubey region). As reported in [8,9] about a third of infected patients required ICU admission. ICU departments need to be well organised to be able to host COVID-19 patients. There is also the need to avoid mixing COVID-19 with other patients in ICUs [10].

In Italy on 15 March, official data reported 24,747 total cases, 20,603 people currently infected, 1809 deaths and 2335 recovered patients.

Among these: 9268 were reported as being treated at home (i.e., not severe illness), 9663 hospitalised, and 1672 admitted to ICUs.

To react to the exponential growth of infected patients requiring hospitalisation, the Chinese government decided to build a large emergency hospital dedicated to COVID-19 patients in a few days. In Italy, the plan was to improve existing structures by extending the number of ICU resources and beds, as well as using dedicated health structures. For instance, the study reported in [11] focuses the necessity of acquiring ICU resources such as ventilators or breathing support devices. Italy has approximately 5200 beds in ICUs, which, by law, are designed to be occupied by patients for the 80% at any given time. Also, these are allocated at a regional level proportionally to its population and are usually managed locally (see Table 1).

Many of such ICU slots were yet occupied by non-COVID-19 patients while as of 15 March 1672 beds are occupied by COVID-19 patients. Considering the infection trend, it is reasonable to predict that the number of ICUs patients will increase. Since ICU resources are limited, there is the need to know in advance how many will be used. Such a decision may regard, for instance, the institution of new ICU beds or the movement of people from one region to another. So, it is crucial to correctly estimate the number of patients that will need ICU treatment [11].

### 3.1. Diffusion Model of COVID-19 

We focus on decision strategy to increase number and structures able to treat COVID-19 patients in intensive units, and thus increasing the number of ICUs. We propose a model able to manage in geographic scale the incoming patients and the ICUs available places. We cover the a need for the development of a predictive model for helping healthcare administrators in managing structure requirements to improve hospitals and patients managements. We extend a compartmental model for epidemiology, and we derive from Italian public data the experimental parameters for simulating the model.

Literature contains many mathematical epidemiological models for studying the dynamics of infectious diseases [12]. These models fall in two main classes: deterministic models that are based on differential equations and stochastic models that are based on Markov processes.

**Figure 1:**
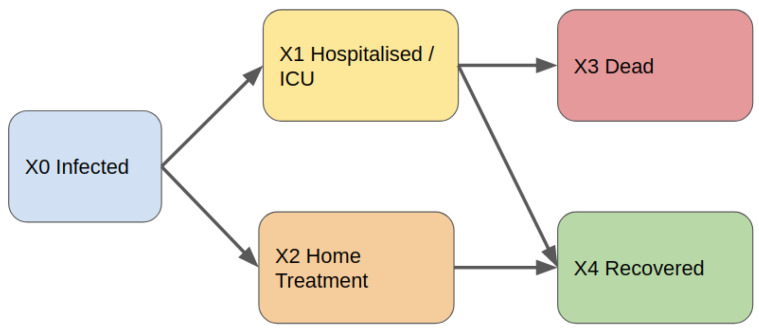
The Compartmental Model. Our model start by considering infected people (X0). A fraction of infected people presents severe symptoms and they need to be hospitalised and treated in ICUs (X1). Diversely, some people may be treated at home (X2) since they do not have severe complications. Treated people has a lethal outcome (X3) while a hopefully large fraction of people is recovered from disease.

We used a discrete-time Markov chain model [13] and we derived the parameters of the model starting from publicly available data, the same described in Section 3. We use as reference a compartmental model which we adapted from the literature [14] (see Figure 1).

**Figure 2:**
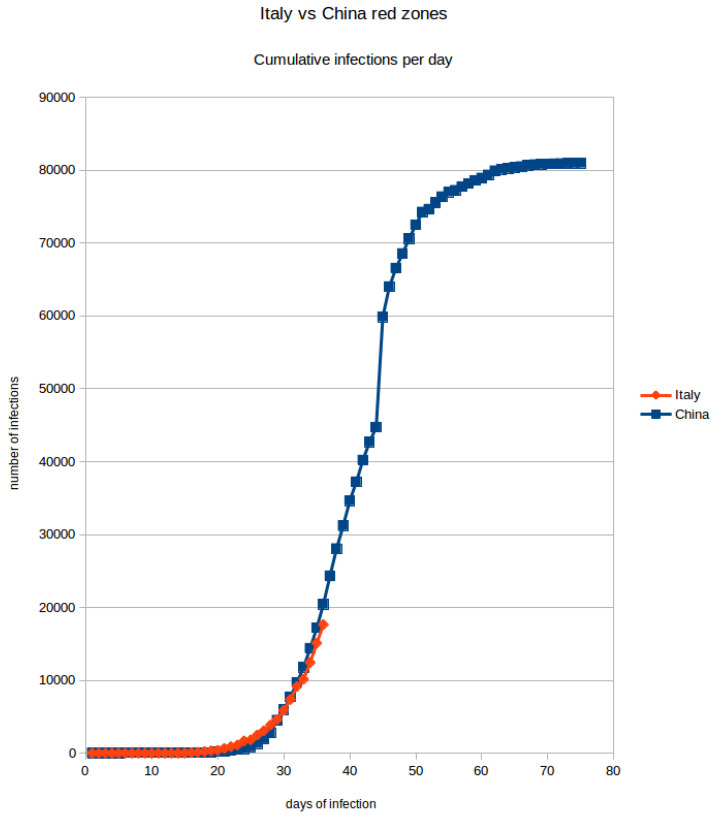
Here the two Italian and Chinese red zones (areas of maximal infection) are compared. On the X axis there are days and on the Y axis there are the total number of cases. The two curves are very similar showing that the initial trend of the infection follows an exponential growth, even though the Chinese government rapidly adopted stringent confinement measures. We can thus expect to observe the same initial infection evolution, before arriving to the logistic portion of the curve.

In Figure 2 the COVID-19 diffusion is reported both for Italy and China red zones (A “red zone” is a geographical area (e.g., city, region, state) of maximal infection for which the government implements special social rules in order to deal with the emergency: typically restriction of citizens’ movements and prohibition to leave or enter the area). We can make the hypothesis of similar trends for different countries (including Italy). Initial exponential growth of the disease is first identified followed by a logistic regression trend as disease spread slows down. In the last phases of the infection, where the curve becomes logistic, diseases have to be treated, continue managing the fraction of patients that require ICUs.

### 3.2. Model at Regional Scale

The Italian National Health Service is organised on a national and regional scale. The central government controls the distribution of resources and services are arranged at a regional scale. There are 19 regions and two autonomous provinces (21 total administrative units). Therefore the ICUs is availability is organised at a regional scale, established by each region. Table 1 summarise current ICU beds availability per administrative units. Patients are admitted into the ICUs of its region, without considering other criteria, such as free beds into ICUs of other regions that may be geographically closer. The access is freely guaranteed costs are mapped to citizen with respect to their regions of residence.

**Figure 3:**
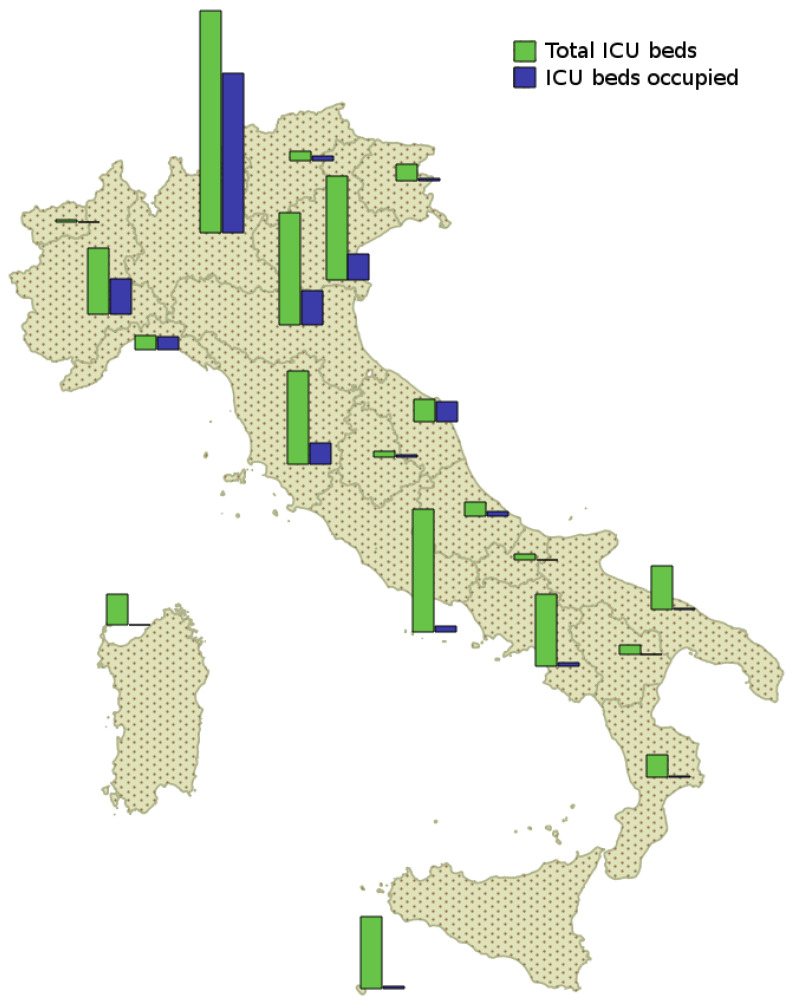
Figure depicts the total number of ICU beds and the number of currently occupied per region. Data is available online on the Italian Civil Protection Department website at https://github.com/pcm-dpc/COVID-19/blob/master/dati-regioni/dpc-covid19-ita-regioni-20200315.csv with licence CC-BY-4.0.

**Figure 4:**
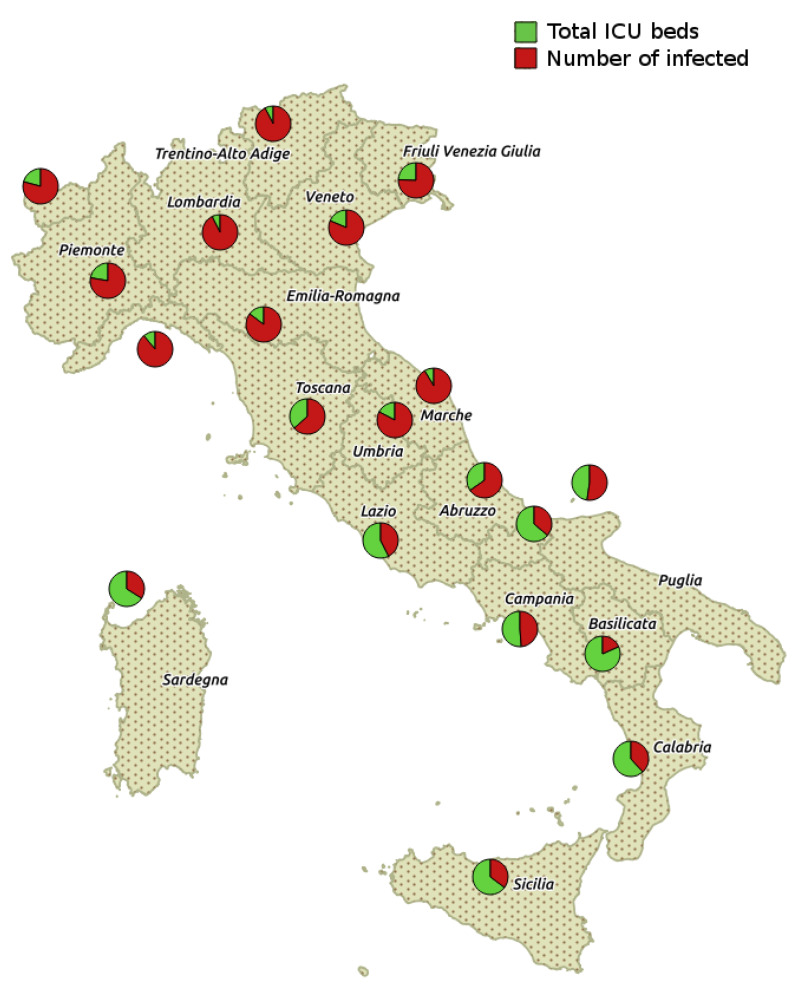
Figure depicts the distribution of COVID-19 infected cases along Italian peninsula. Please note that the number of ICU beds reported are the total and fixed number of available beds for each geographical area. Data is available online on the Italian Civil Protection Department website at https://github.com/pcm-dpc/COVID-19/blob/master/dati-regioni/dpc-covid19-ita-regioni-20200315.csv with licence CC-BY-4.0.

Consequently, some regions may have many available beds while other regions may not. This situation happened in northern Italian regions. Figure 3 shows the distribution of total ICU beds versus occupied ICU beds (i.e., in hospitals) for each region in Italy whereas Figure 4 shows the infected cases for each region.

We compare through our model the management of beds in single regions as compartment and the management of places on a nationwide scale (admitting transfers among regions). Our findings suggest that the management of ICUs beds as a whole across regions may improve the overall availability of free beds for COVID-19 patients.

Figure 3 shows the distribution of total ICU beds versus occupied ICU beds (i.e., in hospitals) for each region in Italy. Figure 4 shows the infected cases for each region.

### 3.3. Model Based ICUs Prediction

**Figure 5:**
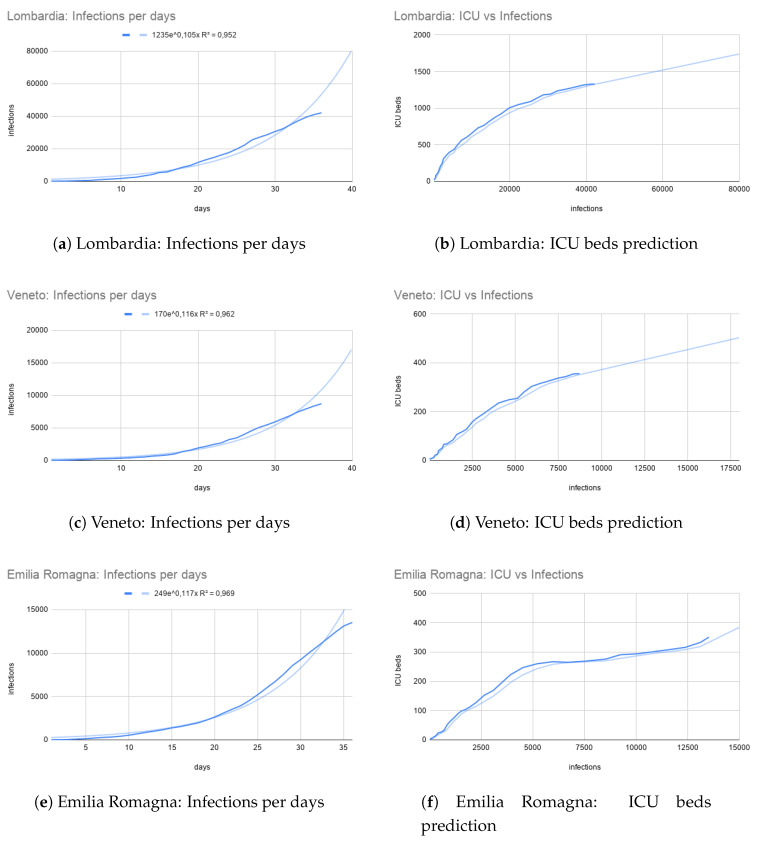
Infections per days (with exponential fitting) and ICU bed vs. Infection (with moving average of modulus 3) graphs for the Lombardia, Veneto and Emilia Romagna northern regions at the date of 30 March.

We associated the number COVID-19 infections with ICU beds occupancy. In particular, the target is to predict the number of ICU beds required for a certain amount of infections in a given region, using COVID-19 trend. This is used to relate infections and ICUs beds (see Figure 5b for Lombardia region). We fitted the datapoints from current COVID-19 infection with an exponential function. By using such a fitting we are able to predict infections (Y axis of Figure 5a) for future days (X axis of Figure 5a). Then, we can use such a number (X axis of Figure 5b) in order to predict the number of ICUs required in the future (Y axis of Figure 5b). These predictions may be used to plan decision about COVID-19 patients and also to reallocate them in different structures.

Furthermore, trends of other Italian regions are reported in Figure 5c and Figure 5d respectively for Veneto region, and Figure 5e,f for Emilia Romagna region. Lombardia, Veneto and Emilia Romagna were the top three regions with an emergency in terms of ICU beds necessity at the date of 30 March (saturated regions).These regions were already reallocating ICU patients in different regions as well as working on plans to free ICU beds or create new ones.

**Figure 6:**
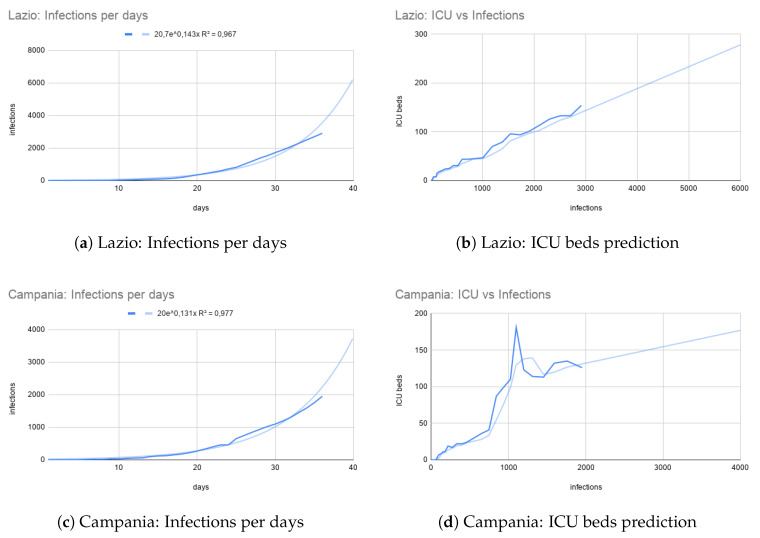
Infections per days (with exponential fitting) and ICU bed vs. Infection (with moving average of modulus 3) graphs for the Lazio and Campania southern regions at the date of 30 March.

Predicting ICU for non-saturated regions. We applied our predicting model to southern Italian regions, when the infection trend was at the beginning (i.e., delayed curve and low numbers) with respect to northern ones. During this phase the ICU beds capacity was under saturation (see Figure 3). We used the predicting model for these regions to early predict saturation phases. In Figure 6a,b a diffusion of disease and relative connections with ICUs requirements are reported and refer to a central Italian region, i.e., Lazio. By using infections vs ICU beds trend, we were able to calculate the number of infections for some time point in the future and derive the number of predicted ICU beds which will be occupied. Similarly, in Figure 6c we report Campania region situation at 30 March as south of Italy representation. In such case note that the government restrictions rapidly adopted allow a slower diffusion of infectious.

Note that in a similar way we map all data for all the 21 Italian regions.

## 4. Conclusions

The emergency of COVID-19 is related to an aggressive virus that diffuses rapidly and strongly stresses the resistance of health structures. Since the COVID-19 related disease require different non-standard protocols, such as the use of respiratory devices, patients treatment is strictly intertwined with the availability of hospital structure resources (e.g., ICU beds). We think that, by using a scalable predictive model, (at regional or district level) may support governments in a better management of the emergency. Finally, the presented model is valid during the exponential growth of the infection. Furthermore, since the swab tests are not available in sufficient numbers to guarantee a wide screening of the population, they are performed only to hubs (i.e., police officers, healthcare personnel) and people dyeing by covid who were previously hospitalized in ICUs. Hence infection numbers are highly underestimated. We claim that such a model could be used in countries where diffusion is still at the beginning, such as US, France, Spain and other European countries (see [15] where virus diffusion trajectories are reported for different countries).

## Figures and Tables

**Table 1:** Distribution of ICU beds in each region at the date of 15 March. Such data could increase in the future due to government investments for the emergency.

Region	Beds	Region	Beds
Piemonte	320	Marche	108
Valle D’Aosta	15	Lazio	590
Lombardia	1067	Abruzzo	73
P.A. Bolzano	48	Molise	30
P.A. Trento	23	Campania	350
Veneto	498	Puglia	210
Friuli Venezia Giulia	80	Basilicata	49
Liguria	70	Calabria	110
Emilia Romagna	539	Sicilia	346
Toscana	450	Sardegna	150
Umbria	30	Italy	5156

## Abbreviations

The following abbreviations are used in this manuscript:

ICU	Intensive Care Unit
